# The Changing Epidemiology of Oropharyngeal Candidiasis in Patients with HIV/AIDS in the Era of Antiretroviral Therapy

**DOI:** 10.1155/2012/262471

**Published:** 2012-08-28

**Authors:** Payal K. Patel, Joshua E. Erlandsen, William R. Kirkpatrick, Deborah K. Berg, Steven D. Westbrook, Christopher Louden, John E. Cornell, George R. Thompson, Ana C. Vallor, Brian L. Wickes, Nathan P. Wiederhold, Spencer W. Redding, Thomas F. Patterson

**Affiliations:** ^1^Department of Internal Medicine, The University of Texas Health Science Center at San Antonio, San Antonio, TX 78229-3900, USA; ^2^South Texas Veterans Health Care System, San Antonio, TX 78229, USA; ^3^Division of Infectious Diseases, The University of Texas Health Science Center at San Antonio, Department of Medicine, 7703 Floyd Curl Drive, MSC 7881, San Antonio, TX 78229-3900, USA; ^4^Department of Comprehensive Dentistry, The University of Texas Health Science Center at San Antonio, San Antonio, TX 78229-3900, USA; ^5^Department of Epidemiology & Biostatistics, The University of Texas Health Science Center at San Antonio, San Antonio, TX 78229-3900, USA; ^6^Department of Internal Medicine, University of California at Davis School of Medicine, Davis, CA 95817, USA; ^7^University of Incarnate Word, San Antonio, TX 78209, USA; ^8^Department of Microbiology and Immunology, The University of Texas Health Science Center at San Antonio, San Antonio, TX 78229-3900, USA

## Abstract

The impact of antiretroviral therapy (ART) on opportunistic conditions in HIV patients continues to evolve. We specifically studied the changing epidemiology of oropharyngeal candidiasis (OPC) in 215 HIV/AIDS patients. Status of yeast colonization was assessed from oral rinse samples, and preliminary yeast identification was made using CHROMagar *Candida* and confirmed with standard microbiological techniques and/or molecular sequencing. Susceptibility to fluconazole was determined by CHROMagar *Candida* agar dilution screening and CLSI broth microdilution. 176 (82%) patients were colonized and 59 (27%) patients had symptomatic OPC. *Candida albicans* was the most prevalent species, though *C. glabrata* and *C. dubliniensis* were detected in 29% of isolates. Decreased fluconazole susceptibility occurred in 10% of isolates. Previous ART reduced the risk of OPC, while smoking increased the risk of colonization. Oral yeast colonization and symptomatic infection remain common even with advances in HIV therapy. *C. albicans* is the most common species, but other yeasts are prevalent and may have decreased susceptibility to fluconazole.

## 1. Introduction

*Candida albicans* is a frequent component of oral ecology found in up to 75% of humans [1]. In immunocompromised patients, *Candida* species can cause a multitude of disease manifestations ranging from mild oral disease to disseminated candidiasis. Diagnosis and treatment of disease caused by *Candida* species is especially important in HIV/AIDS patients who, despite the advent of antiretroviral therapy (ART), continue to suffer significant *Candida *associated morbidity  [2, 3].

Of particular concern is oropharyngeal candidiasis (OPC), an opportunistic yeast infection of the soft and hard palates, pharynx, tongue, and buccal mucosa. OPC can present as erythematous patches or white, removable curdlike lesions and is often the first clinical sign of underlying HIV infection [3]. OPC is seen with a higher prevalence in patients with CD4+ counts below 200/mm^3^ or a high viral load (>10,000 copies/mL) [2]. Since the advent of ART in the 1990s, there has been a significant decrease in HIV-related opportunistic infections, including OPC [4]. However, the epidemiology of yeast colonization and infection in the era of ART is not well established.

OPC caused by *C. albicans* is generally managed by judicious use of fluconazole. A rise in resistant organisms may be due to prolonged or frequent treatment with azoles. Because certain *Candida* species are either intrinsically resistant to fluconazole (*C. krusei)* or can rapidly develop resistance (*C. glabrata* and *C. dubliniensis)*, an epidemiologic shift of *Candida *species could significantly impact the utility of fluconazole as empiric treatment for candidiasis in patients with HIV/AIDS [5]. This study examined the changing epidemiology of OPC as HIV is increasingly approached as a chronic disease and ART therapy is recognized as the standard of care for HIV. We investigated postulated epidemiologic factors for OPC colonization and symptomatic infection in a population of patients with HIV/AIDS and CD4+ counts under 200/mm^3^.

## 2. Methods

### 2.1. Patient Recruitment

In this study, 215 HIV-positive patients were recruited from the Family Focused AIDS Clinic and the South Texas Veterans Immunosuppression Clinic, both located in San Antonio, Texas. Data was reported from baseline visits of a four year prospective longitudinal observational study. Inclusion criteria involved HIV/AIDS diagnosis, a CD4+ count ≤200/mm^3^ or an active clinical diagnosis of OPC, and a minimum age of 18. Informed consent was obtained, and procedures were in accordance with the Institutional Review Board of The University of Texas Health Science Center at San Antonio (UTHSCSA) and the Research and Development Committee of the South Texas Veterans Health Care System. Patients with a new diagnosis of HIV received standard ELISA screening with Western Blot confirmation at University Hospital Laboratory, (University Health System, San Antonio, TX, USA). The Lymphocytic Lab at UTHSCSA was utilized to count CD4+ cells. Upon enrollment, participants were interviewed by clinicians who collected epidemiologic data. Past medical history was available via the university and VA medical records.

### 2.2. Oral Rinse Samples and Antifungal Susceptibility

Oral samples were taken using buccal swabs and by having patients swish and spit 10 mL of sterile water [5]. Samples were plated using CHROMagar *Candida* (CHROMagar Company, Paris, France) media with fluconazole (0, 8 and 16 *μ*g/mL) to screen for susceptibility of isolates to fluconazole. Susceptibility was confirmed using standard Clinical Laboratory Standards Institute (CLSI) methodology [6]. CHROMagar *Candida* was prepared according to the manufacturer's instructions. Color patterns remained unchanged on media with or without fluconazole [7].

### 2.3. Microbiological Characterization

Chromogenic medium containing chloramphenicol (0.5 g/L) and agar (15 g/L) was dispensed into 100-mm-diameter petri dishes and stored at 4°C. CHROMagar *Candida*-specific color patterns were used for presumptive identification. Yeasts were further characterized by germ tube analysis after incubation in human serum at 37°C for 3 hours, and metabolic characterization with API 20C (bioMérieux, Marcy-l'Etoile, France) [5].

### 2.4. Molecular Characterization

DNA extraction from yeast present in the oral rinse sample was performed using Prepman reagent (Applied Biosystems, Courtaboeuf, France) or Qiagen QIAamp DNA extraction kits (Qiagen, Valencia, CA, USA) [8]. *Candida-*specific block-based PCR on DNA extracted from patient swish samples was amplified with N18 primers targeting a conserved region of the 18 s rRNA gene [8]. *Candida *isolates from patients were obtained by microbiological methods and further identified to the species level by standard sequencing reactions utilizing ITS1-NL4 primers (Advanced Nucleic Acids Core Facility, UTHSCSA) [9].

### 2.5. Data Management

Multisite longitudinal patient data and microbiological data were managed with a secure, password protected data entry system. This system, the Informatics Data Exchange and Acquisition System (IDEAS) is a web-based Oracle database that can be queried for statistical analysis of molecular and clinical epidemiological data. Molecular and culture data were entered in a blinded approach.

### 2.6. Statistical Methods

Descriptive statistics for demographic features were compared using Student's *t*-test and Kruskal-Wallis test for continuous data and Pearson's Chi-Squared test or Fisher's exact test for categorical data. Logistic models were constructed to calculate the odds ratios that a certain covariate would lead to colonization, OPC or decreased susceptibility to fluconazole. Covariates included CD4+ count, viral load, age, race, gender, history of smoking, esophageal candidiasis, antifungal use, ART, and denture use. All statistical calculations were performed using SAS version 9.2 for Windows (SAS Institute Inc, Cary, NC).

## 3. Results

### 3.1. Demographic Data

Two hundred and fifteen patients diagnosed with HIV were enrolled in this longitudinal study. At visit one, 176 patients were colonized with one or more oral yeasts and 39 were non-colonized. Initially, 59 of 215 (27%) patients presented with symptomatic OPC. The cohort of 215 patients was 89% male and 57% identified themselves as Hispanic. The average age was 43 ± 9 years, and the average years since diagnosis of HIV was 8 ± 7 years. Sixty-nine percent of patients were receiving ART. Of those colonized, 66% (117/176) were receiving ART (Table 1) and of those presenting with OPC, 51% (30/59) were receiving ART.

Of all patients enrolled, 8% (17/215) had diabetes, which is often associated with OPC, especially when uncontrolled [10]. Seventy percent (151/215) of those enrolled had a positive smoking history and 21% of all patients used dentures. Thirty-seven percent (79/215) of patients reported previous antifungal use. CHROMagar screening and CLSI susceptibility testing identified 7% (16/215) of patients who grew yeast species with decreased susceptibility (MIC >8) to fluconazole. Three of these patients grew more than one yeast species with decreased fluconazole susceptibility.

The CD4+ T cell counts for all patients ranged from 1 to 392 cells/mm^3^. The median CD4+ for patients with OPC was 67.5 (IQR: 17.5–135.5), and for colonized patients was 90 (IQR: 35–152). Median viral load for those patients colonized was 26,600 copies/mL (IQR: 425–153000), and for those with OPC was 50,350 copies/mL (IQR: 4229–219000).

Previous antifungal use was protective against colonization (OR: 0.44 (95% CI: 0.22–0.91); *P* value: 0.026) while a positive tobacco history was associated with an increased susceptibility to colonization (OR: 4.07 (95% CI: 1.18–14.08); *P* value: 0.027). ART was also protective regarding developing OPC (OR: 0.36 (95% CI: 0.18–0.72); *P* value: 0.004) as was age (OR: 0.94 (95% CI: 0.91–0.98); *P* value: 0.006). The risk of patients harboring yeasts with decreased susceptibility to fluconazole was increased with a history of esophageal candidiasis (OR: 7.16 (95% CI: 1.22–41.96); *P* value: 0.029), or wearing dentures (OR: 3.06 (95% CI: 1–9.37); *P* value: 0.049). (Table 2).

### 3.2. Clinical and Microbiological Data

*Candida albicans *was the most frequently isolated species, found in 137/222 (62%) of all isolates from colonized patients. Non-*albicans* yeasts were also frequently detected, including *C. glabrata* in 17% of all isolates from colonized patients, *C. dubliniensis* in 12%, *C. tropicalis* 5%, *C. krusei* 2%, and *C. parapsilosis with* 1%. CHROMagar screening and CLSI testing revealed decreased fluconazole susceptibility (MIC >8 *μ*g/mL) occurred in 16/176 (9%) of patients with microbiologically confirmed colonization or infection, and identified 6 isolates that exhibited frank resistance to fluconazole (MIC ≥64).

 The MIC_50_ and MIC_90_ of *C. albicans *were 0.125 and 1, respectively. For *C. glabrata* the MIC_50_ and MIC_90_ were 8 and 32. Nine of the 59 (15%) OPC patients carried yeasts with decreased fluconazole susceptibility. These nine patients grew 12 yeasts with decreased susceptibility to fluconazole, 75% were *C. glabrata,* and 25% were identified as *C. albicans. *Three of these infections resulted from mixed colonization with *C. albicans *and* C. glabrata.*

Favorable response to fluconazole occurred in 58 of 59 (98%) patients with symptomatic infection after fluconazole therapy. Favorable responses occurred in all 50 OPC patients with susceptible isolates, although one patient required an increased dose (400 mg) for response. Of 9 OPC patients with decreased fluconazole susceptibility, 8 responded (89%) although 2 (22%) either received doses of 200 mg or more per day as initial therapy or required escalation of doses for clinical improvement.

## 4. Discussion

Comparable with studies done internationally, primary infections susceptible to fluconazole were largely caused by *C. albicans* in this study [2, 3, 10–13]. Of the non*-albicans *species identified in this study, *C. glabrata* was most frequently isolated followed by *C. dubliniensis*. Increased prevalence of *C. dubliniensis* in the last decade may be due to increased identification of the species, which can be mistaken phenotypically as *C. albicans* [7]. Regarding OPC with decreased susceptibility to fluconazole, *C. glabrata* was the most prevalent species. *C. glabrata *is well known for its frequent resistance to azole antifungal agents, and it has been shown that *C. glabrata* often rapidly acquires fluconazole resistance with azole exposure and can upregulate drug efflux pump genes with as little as four days of in vitro fluconazole exposure [14].

Since the introduction of fluconazole therapy, treatment of OPC has become simple, low cost, and efficacious [5]. Current guidelines note that for AIDS patients with a history of recurrent OPC, 200 mg/day of prophylactic fluconazole is acceptable and does not appear to lead to resistance [15]. In our study, previous antifungal use was associated with decreased odds (OR: 0.44, *P* = 0.026) of having increased colonization and had no impact on resistance. Our data show that the propensity of patients having fluconazole resistant yeasts was increased by having previously had esophageal candidiasis or wearing dentures.

Interestingly, the data suggests that denture use is an important risk factor for growing species of *Candida *with decreased fluconazole susceptibility. The odds ratio in patients with dentures of growing yeast with decreased susceptibility to fluconazole was 3.06 (*P* = 0.049). The surface irregularities of acrylic resin are a factor in the entrapment of microorganisms, and the resulting biofilm formation may become a protective reservoir for oral yeast [11]. Increasing age has been associated with higher incidence of *C. glabrata*, and dentures are generally worn by older individuals [16]. As oral flora changes with age, ill-fitting dentures may irritate the oral cavity and resulting OPC may be more likely to be caused by *C. glabrata*. Fifty-five percent (12/22) of isolates with decreased susceptibility to fluconazole in this study were *C. glabrata*, and 27% (6/22) were *C. albicans. *This is consistent with the majority of reports of fluconazole-resistant species in HIV patients [10, 13]. Emergence of *C. glabrata* as a pathogen in oral candidiasis may have significant clinical implications as conventional doses of fluconazole are often ineffective to clear this infection. CHROMagar based yeast screening for reduced fluconazole susceptibility and CLSI MIC testing identified 16 patients as having yeast with reduced fluconazole susceptibility. Indeed, 6 isolates had MICs of 64, emphasizing the importance of monitoring susceptibility in this population. Examination of the development of yeast resistance in these patients over the full duration of this study is warranted.

## 5. Conclusions

In summary, oral yeast colonization and symptomatic infection remain common in patients with HIV/AIDS, even with ART. *C. albicans* is the most common species isolated in patients. Overall, clinical outcomes with fluconazole were favorable although higher doses were needed for response, particularly in patients with reduced fluconazole susceptibility. Yeasts other than *C. albicans*, including *C. dubliniensis and C. glabrata* were commonly isolated and can be significant species in the development of fluconazole resistance [10, 11, 15]. Oral yeast colonization and the possibility of resistant yeasts should be considered in patients with HIV who have risk factors for invasive infection or who have symptoms that persist after fluconazole therapy. Our results emphasize the need for continued awareness of OPC as a major cause of morbidity in HIV populations.

## Figures and Tables

**Table 1:** Patient demographics of 215 subjects by colonization status.

	Colonized		
	Yes (*N* = 176)	No (*N* = 39)	*P*-value
Age, mean (SD)	42.7 (8.9)	42.3 (8.5)	0.779^1^
Male, *n* (%)	157 (89.7)	34 (85)	0.393^2^
White, *n* (%)	54 (30.9)	7 (17.5)	0.134^3^
Non-Hispanic, *n* (%)	82 (46.9)	11 (27.5)	0.026^2^
Years since diagnosis of HIV/AIDS, mean (SD)	8.4 (6.9)	6.4 (6.5)	0.086^4^
Diabetic, *n* (%)	15 (8.5)	2 (5)	0.745^3^
History of Smoking, *n* (%)	127 (72.1)	24 (60)	0.117^2^
Uses dentures, *n* (%)	42 (23.8)	3 (7.5)	0.018^3^
Thrush present, *n* (%)	59 (33.5)	0 (0)	<0.001^3^
History of esophageal *Candida*, *n* (%)	8 (4.5)	3 (7.5)	0.433^3^
Decreased susceptibility to fluconazole, *n* (%)	16 (9.1)	0 (0)	0.047^2^
On ART therapy, *n* (%)	117 (66.4)	31 (79.5)	0.145^2^
History of antifungal use, *n* (%)	59 (33.5)	20 (50)	0.054^2^
CD4 count, mean (SD)	102.8 (80.3)	84.9 (67.8)	0.283^4^
Viral load, mean (SD)	607142.5 (3442957.1)	103189.7 (161265.9)	0.588^4^

^1^ *F* test.

^2^Pearson's *χ* ^2^ test.

^3^Fisher's exact test.

^4^Kruskal-Wallis test.

**Table 2:** Logistic models of different epidemiologic factors impacting OPC, colonization, and decreased susceptibility to fluconazole.

Model^1^	Odds ratio (CI)	*P* value
Colonization status^2^		
Smoker	4.07 (1.18, 14.08)	0.027
History of antifungal use	0.44 (0.22, 0.91)	0.026
OPC presence^3^		
Age	0.94 (0.91, 0.98)	0.006
On ART therapy	0.36 (0.18, 0.72)	0.004
Decreased susceptibility to fluconazole^1^		
History of esophageal *Candida*	7.16 (1.22, 41.96)	0.029
Denture use	3.06 (1, 9.37)	0.049

^1^All models are logistic models constructed using forward model selection on the covariates CD4 count, viral load, age, race, ethnicity, gender, history of smoking, diabetes status, history of esophageal *Candida*, denture use, on ART, and the history of antifungal use.

^2^The dependent variable is colonized (yes/no).

^3^The dependent variable is presence of OPC (yes/no).

^4^The dependent variable is decreased susceptibility to fluconazole (yes/no).
